# Meta-analytical methods to identify who benefits most from treatments: daft, deluded, or deft approach?

**DOI:** 10.1136/bmj.j573

**Published:** 2017-03-03

**Authors:** David J Fisher, James R Carpenter, Tim P Morris, Suzanne C Freeman, Jayne F Tierney

**Affiliations:** 1 London Hub for Trials Methodology Research, MRC Clinical Trials Unit, University College London, London, UK; 2 Department of Medical Statistics, London School of Hygiene and Tropical Medicine, London, UK

## Abstract

Identifying which individuals benefit most from particular treatments or other interventions underpins so-called personalised or stratified medicine. However, single trials are typically underpowered for exploring whether participant characteristics, such as age or disease severity, determine an individual’s response to treatment. A meta-analysis of multiple trials, particularly one where individual participant data (IPD) are available, provides greater power to investigate interactions between participant characteristics (covariates) and treatment effects. We use a published IPD meta-analysis to illustrate three broad approaches used for testing such interactions. Based on another systematic review of recently published IPD meta-analyses, we also show that all three approaches can be applied to aggregate data as well as IPD. We also summarise which methods of analysing and presenting interactions are in current use, and describe their advantages and disadvantages. We recommend that testing for interactions using within-trials information alone (the deft approach) becomes standard practice, alongside graphical presentation that directly visualises this.

Meta-analysis of participant level treatment-covariate interactions raises additional complications that do not affect either standard meta-analysis or single trial interaction analysis alone. These complications are often overlooked by reviewers. The issues are independent of other aspects of meta-analysis, such as the choice of one stage or two stage model fitting, or whether aggregate data or individual participant data (IPD)^1^ is used. The three titular approaches discussed in this paper are derived from two independent quantities referred to in recent IPD literature as across-trial and within-trial interactions.^2^ ^3^ We have used three descriptive terms to be memorable and to avoid ambiguity. “Daft” (meaning absurd or preposterous) refers to estimation of the across-trial interaction alone. “Deluded” (meaning misleading or deceiving) refers to an estimation of both the across-trial and within-trial interactions combined. “Deft” (demonstrating skill or cleverness) refers to estimation of the within-trial interaction alone. As their monikers suggest, daft and (to a lesser but non-negligible extent) deluded approaches are at risk of bias^4^—the extent of which is typically unknown—whereas deft approaches are not.

**Summary points**

Meta-analysis is often the only way to reliably detect whether treatment benefit differs between groups of participants—that is, to detect interactions between treatment efficacy and participant characteristics
Of three general approaches, we advocate the deft approach, which avoids the risk of ecological bias over the deluded and daft approaches, which do not
A systematic review of recently published meta-analyses of individual participant data shows that 89% of interaction analyses either used a deluded method (23%), or did not report sufficient details (66%) to tell which approach had been used. Further, graphical presentation often hindered appreciation of key data features
These findings indicate poor appreciation of analysis and reporting issues surrounding interactions in the research community
Where suitable data were reported in published systematic reviews, reanalysis showed that use of the different approaches can yield inconsistent results
Testing for interactions using deft methods, and using a graphical presentation that directly visualises this, should become standard practice

## How to analyse and present participant level interactions data from a meta-analysis: daft, deluded, or deft approach

Our illustrative example is a systematic review and IPD meta-analysis relating to the care of patients with acute stroke, which compared a strategy of early supported hospital discharge (ESD) to conventional hospital services and discharge arrangements.^5^ ESD reduced the mean duration of initial hospital stay. The authors investigated whether this effect varied according to whether a participant had a carer present—that is, whether there was an interaction between treatment and presence of a carer. Here, we describe the results from this published IPD meta-analysis using three approaches we previously identified.^2^

### Daft approach (across-trial interaction alone)

This approach can be visualised by plotting the mean difference in the length of hospital stay (ESD *v* standard care) for each trial against the proportion of trial participants who had a carer present. Fitting a meta-regression^6^ to the data points, we find that as the proportion of trial participants with a carer present increases, the benefit of ESD decreases (P<0.001, web fig 1). This analysis focuses solely on how the overall effect of ESD varies across trials with different proportions of carers present, relying on an aggregated summary of the carer information for each trial rather than considering whether the effect for an individual participant varies according to the presence of a carer. Such an analysis is easily confounded and is at risk of ecological bias, whereby interactions at the aggregate (or “ecological”) level might not reflect the true interaction at the individual participant level.^4^ Given the available data, this is a daft approach.

### Deluded approach (within-trial and across-trial interactions combined)

This approach is presented in figure 1. The left panel shows an attempt to make better use of the data by carrying out a meta-analysis within the subgroup of participants who had a carer present, and another in those who did not. The right panel shows a simpler alternative presentation of this type of analysis that is often seen in the literature. The interaction test compares the treatment effects in the two carer subgroup level meta-analyses, but can also be extended to explore trends in effect across more than two ordered categories, such as stage of disease. As in the original review,^5^ this approach suggests that the presence of a carer does not modify the effect of ESD (P=0.39). However, consider how the data is actually being used: trial data is split into participant subgroups, treatment effects are combined within these subgroups, and are then compared across subgroups. This process combines within-trial and across-trial interaction estimates.^3^ The analysis is again subject to ecological bias, although the addition of across-trial information can provide a gain in power. How the treatment effect varies at the individual participant level (that is, the within-trial interaction) could be exaggerated or masked by the across-trial interaction; we are at risk of being deluded. In the large Montreal trial,^w87^ all participants had a carer, and hence this trial can contribute only an across-trial interaction (web fig).

**Figure f1:**
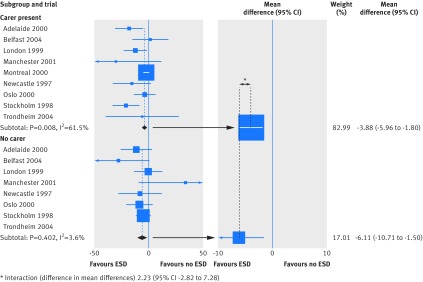
**Fig 1** Use of a deluded approach to analyse and present interactions in meta-analysis , illustrating how the effect of an early supported hospital discharge (ESD) strategy might vary by whether a carer is present.^5^ Left panel presents the effect of ESD for each subgroup within each trial, but ordered by subgroup; and right panel presents just the meta-analysed effects for each subgroup. The difference between the effects in right panel gives a deluded analysis (mean difference of 2.23, 95% confidence interval −2.82 to 7.28, P=0.39). Sizing of squares are in proportion to the inverse of the variance of the estimates. Note that the subgroup meta-analysis estimates do not match exactly those originally reported, because we used a fixed effect model for simplicity, rather than the random-effects model used by the original authors. See also web appendix A3 for references of studies and appendix B2 for details of statistical reanalysis

### Deft approach (within-trial interaction alone)

This approach adheres to the underlying principles of meta-analysis by assessing the effect of interest as measured within each relevant trial. The left panel in figure 2 shows the same data as those in figure 1, but rearranged so as to demonstrate this approach. We test for an association between the effect of ESD and presence of a carer within each trial, instead of testing for an association across carer subgroups. These interactions, interpretable as the difference in treatment effect for an individual participant with a carer present compared to one without, can be combined and presented by use of standard meta-analytical techniques (fig 2, right panel).^7^ In this case, the results show that having a carer present increases the effect of ESD on duration of hospital stay relative to standard care (P=0.077). The right panel in figure 2, however, also shows some visual evidence of heterogeneity (confirmed statistically (I^2^=45%)) among the interaction estimates, suggesting that presence of a carer might not be a wholly reliable indicator of ESD efficacy. Appropriately, the Montreal trial is not included in this deft analysis, because a within-trial interaction is not estimable.

**Figure f2:**
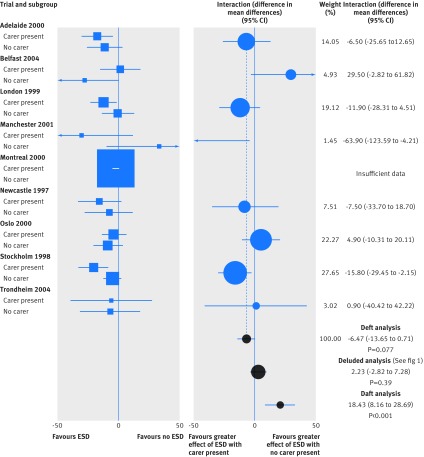
**Fig 2** Use of a deft approach to analyse and present interactions in meta-analysis , illustrating how the effect of an early supported hospital discharge (ESD) strategy might vary by whether a carer is present.^5^ The left panel again presents the effect of ESD for each subgroup within each trial, but now ordered by trial. The right panel shows the interactions between the effect of ESD and presence of a carer for each trial, along with a meta-analysis of the interaction estimates (mean difference –6.64, 95% confidence interval –13.65 to 0.71, P=0.77; heterogeneity of interaction estimates: Q=12.8, df=7, I^2^=45%). Daft and deluded interaction estimates are presented alongside for comparison. Squares are used to depict treatment effect and circles the interaction effects, with sizing in proportion to the inverse of the variance of the estimates

## Systematic literature review of published interactions between treatment effects and participant level covariates

Having highlighted what is at stake, we present the results of our systematic review of the literature. We searched Medline (2011-14) for IPD meta-analyses of treatment efficacy that included at least one investigation of interaction between treatment and a participant level covariate (web appendix A). Our search returned 184 unique results, of which 80 were eligible. However, one ineligible study cited two additional IPD meta-analyses, giving a total of 82 eligible results. Table 1 summarises the analysis and presentation methods used.

**Table 1 tbl1:** Systematic literature review: presentation and analysis of treatment-covariate interactions

Primary method of analysis	No (%) of meta-analyses	Primary method of presentation					
		Forest plot by subgroup only	Forest plot by subgroup and trial	Forest plot of interactions	Kaplan-Meier plots by subgroup	Line plots (for continuous covariates)	No plot
Total No (%) of meta-analyses	82 (100)	35 (43)	7 (9)	2 (2)	7 (9)	3 (4)	28 (34)
Deft*	2 (2)	—	—	—	—	—	2 (2)
Deluded*	19 (23)	13 (16) §	3 (4)	—	—	—	3 (4)
Unclear†	54 (66)	20 (24)	3 (4)	2 (2)¶	6 (7)	2 (2)	21 (26)
Descriptive only‡	7 (9)	2 (2)	1 (1)	—	1 (1)	1 (1)	2 (2)

Primary methods of analysis and presentation were considered to be those either described as such, or appearing first in the article. Generally, only one method was used per review; the exceptions are detailed below.

*A statistical test was used that satisfies the definition of a deft or deluded approach as given in this article, including one stage models.

†A statistical test was done (including one stage model fitting), but insufficient details were given for it to be definitely described as a daft, deluded, or deft analysis.

‡Results were presented by subgroup, but no statistical tests of interaction were reported or implied.

§Two reviews^w54, w68^ also stated that a deft analysis might be used for sensitivity; one did in fact present such a plot in an additional publication (see ¶).

¶Another review^w68^ also presented a forest plot of interactions in their Cochrane Database review,^8^ but not in their peer reviewed journal article.

Efforts to advocate a deft approach^2^ ^9^ (2% of analyses) seem to have been largely unheeded, while the deluded approach remains popular (23% of analyses). Encouragingly, no daft analyses were explicitly reported. However, in our sample, the majority of reviews (66% of analyses) provided insufficient information to identify whether a daft*, *deluded, or deft analysis was carried out. This includes both so-called one stage modelling approaches (83% of insufficiently described analyses),^10^ which could be either deluded**or**deft,^2^ ^3^ and other commonly used but deluded approaches.^5^ ^10^ Given that such results might be used to inform clinical practice, this lack of detail is disturbing.^11^

The majority of reviews (56% of 82) tested for interaction exclusively by calculating the treatment difference between just two participant level subgroups, even where the underlying data were continuous or ordinal. Such collapsing of data is known to be suboptimal, both in terms of power and bias, as well as biologically implausible.^12^ Considering the low power inherent in interaction testing, this suggests that the analysis of interactions could be better planned.^13^

In our sample, other than presenting no plot at all (34% of reviews), the most common presentational approach (43%) by far was to present summary treatment effects within each covariate subgroup (fig 1, right panel). As illustrated above, this approach invites readers to combine within-trial and across-trial interaction estimates, potentially deluding them about the strength of the evidence. A minority of reviews (9%) expanded on this, presenting data by trial within each subgroup (fig 1, left panel). This approach has more to commend it, because more information is displayed; indeed, it provides sufficient data to allow the reanalyses reported in the next section. 

Nevertheless, we prefer that treatment effects by subgroup (fig 2, left panel) or the resulting interactions (fig 2, right panel) are plotted within each trial, because this corresponds more directly with a deft analysis. Disappointingly, only 2% of reviews adopted such an approach^w19, w55^ by presenting within-trial interactions and meta-analyses of these interactions together in a forest plot (fig 2, right panel). Two further reviews^w54, w68^ used a deluded approach primarily, but stated an intention to carry out an additional deft analysis for sensitivity (see footnotes to table 1).^8^

## Reanalysis of published data from systematic literature review 

Six meta-analyses from our review^5, w21, w28, w38, w47, w61^ presented sufficient data to allow reanalysis of 31 interactions, providing useful insight into the implications of using the three approaches. This reanalysis (web appendix B) is itself a demonstration that aggregate data, albeit in this case derived from IPD, can be analysed by any approach. Our results suggest that, in practice, only deluded or deft analyses are used. However, deluded analyses combine information from daft and deft analyses, which we found to be poorly correlated (fig 2), demonstrating why a deluded analysis might be misleading. In practice, we found deluded analyses were more likely to be significant at the 5% level than deft analyses (16% *v* 6%).

**Figure:**
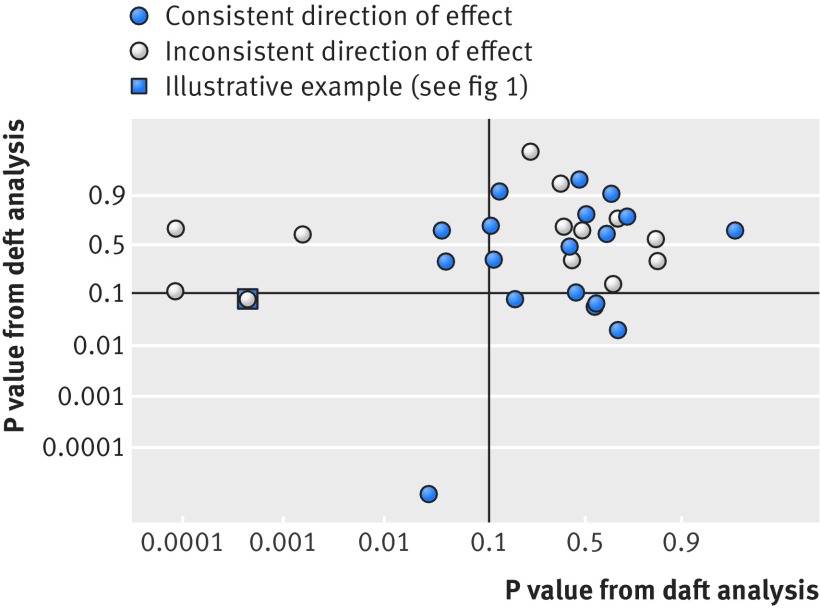
**Fig 3** Scatter plot (logit scale) of P values from 31 deft reanalyses (see web appendix B) of treatment-covariate interactions against the corresponding daft reanalyses. Added lines are at P=0.1; arguably a suitable level of significance for an interaction test for which a trial was not powered

We next made a descriptive assessment of levels of agreement between effect estimates from deluded and deft analyses.^14^ Our results (fig 4) show that deluded analysis did not result in a systematic bias in effect size, although any individual analysis could differ from its deft equivalent by up to 20% in either direction. There seemed to be two distinct patterns of discrepancy. Firstly, the effect size as estimated from the deluded analysis could be substantially larger than that from the deft analysis, as also demonstrated by our illustrative example (fig 2). This is likely a result of differences in the distribution of participant characteristics across trials,^15^ inducing a strong daft effect that confounded the deluded result. Secondly, the effect size as estimated from the deluded analysis could be comparable to that from the deft analysis, except that the deft analysis might not reach significance but the deluded analysis does, by virtue of gaining power from the daft.

**Figure f4:**
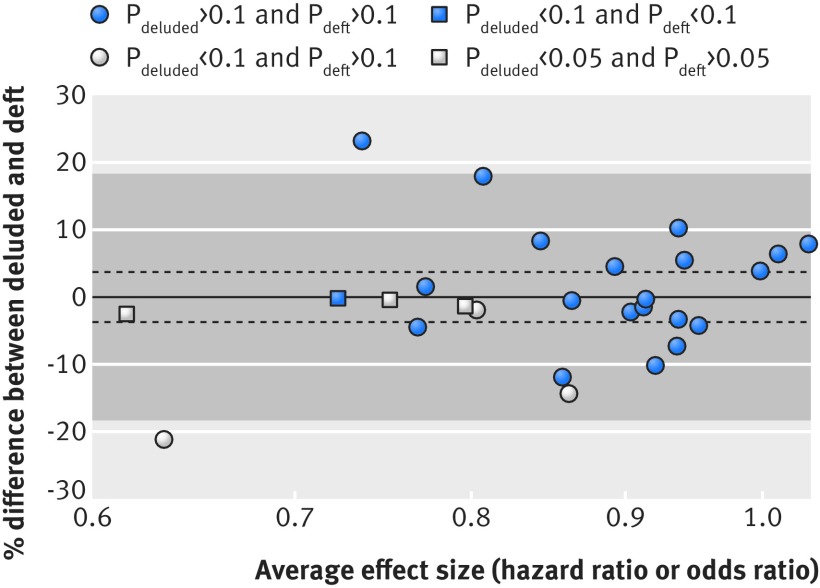
**Fig 4** Bland-Altman^14^ plot showing level of agreement between treatment-covariate interactions from deluded and deft analyses. Thirty one interactions were reanalysed, but only 26 with outcomes measured by hazard ratios or odds ratios were plotted. The remaining five interactions, including our illustrative example, could not be included since their outcomes were measured by mean differences and were hence incompatible. Treatment-covariate interactions (measured on the log scale) might have a positive or a negative sign, but in this plot they have all been set to negative. Hence, differences in interaction effects below the zero line represent cases where a deluded analysis gives a result in the same direction as, but more extreme than, the equivalent deft analysis, and vice versa. Shaded area=Bland-Altman 95% limits of agreement^14^; solid line represents mean difference (bias); dashed lines are 95% confidence intervals around the mean difference

## Recommendations for analysis and presentation of interaction data

Poor reporting and presentation, particularly when combined with inappropriate methods, is a key shortcoming of interaction analyses. Given that a deft analysis is at the least risk of bias, and can be clearly presented and readily interpreted, it should be the preferred approach for assessing participant level interactions aimed at influencing clinical practice. This can be achieved (eg, using Stata^16^) with either IPD^17^ or aggregate data; although the aggregate data requires treatment effects to be reported by subgroup or similar.^18^ If these are not available, but interaction analysis remains an important component of the research project, we recommend requesting suitable aggregate data, or the full IPD, from the trial investigators. Going forward, we recommend that trial investigators routinely report all by-subgroup effect estimates generated by trial analyses.

Plotting treatment effects in each participant group for each trial alongside a forest plot of the within-trial interactions (fig 2) focuses attention on those effects and their association with the interaction estimates. Use of circles instead of squares for the interaction estimates^2^ (fig 2, right panel) helps distinguish such plots from those of main treatment effects. If space is limited, the summary within-trial interaction effect^2^ for a series of covariates can be displayed on one forest plot—for example, figure 3 in reference 17, which may be accompanied by a more detailed plot for those analyses meriting further exploration—for example, web figure 2 in reference 17). Further detail, to provide greater clarity for what is currently a less familiar approach, could be provided by a plot similar to the entirety of figure 2.

Although a forest plot of treatment effects by covariate subgroup (eg, fig 1, right panel) might be considered clinically useful, the visual and statistical comparison of such effects in the meta-analysis context remains deluded. This could invite claims regarding the efficacy of treatments for particular participant subgroups without appropriate regard to a test for interaction.^19^ Many reviewers continue to use deluded analysis methods—possibly because they have been widely used by well respected groups for many years^10^—which risks misleading conclusions and potentially inappropriate clinical recommendations. 

However, within-trial (deft) identification of interactions is relatively rare,^2^ probably in part because of low power. If the across-trial (daft) interaction provides greater power,^15^ then both sources combined (deluded) could substantially improve the power of the interaction estimate over within-trial information alone, albeit with an increased risk of ecological bias. This may be particularly true where some trials, such as Montreal^w87^ in our illustrative example,^5^ only contribute data to one subgroup and therefore cannot be included in a deft analysis. Our reanalysis of the published data suggests that ecological bias might be present in deluded analyses even when based on a set of trials identical to that used in the deft (data not shown). Hence, although a deluded approach might have a role in exploratory analyses or hypothesis generation, we strongly recommend that this intention be made explicit. Moreover, in such cases, within-trial, across-trial, and combined interactions should be presented separately so that readers will not be deluded. Rather, they can make their own judgments about the usefulness or otherwise of the daft estimate.

The two stage approach we recommend here has been criticised for being overly simple, inflexible, and possibly underpowered. Certainly, one stage models fitted to the entire dataset simultaneously allow for greater complexity; but an assessment of their relative power is confounded by their ability to produce either deluded or deft results depending on the separation of within-trial and across-trial effects.^2^ ^3^ Unfortunately, we have found that the details required are rarely provided to judge whether models have been correctly specified so as to accomplish this.^13^ Furthermore, it is not obvious how best to present graphically the results of a one stage model. We would recommend that it be used primarily for inference, while an additional two stage model (which can produce only deft results) is used as the basis of the forest plot. This approach has previously been suggested in the context of main treatment effects.^20^

Our example (fig 1) shows how to proceed with a binary covariate. Categorical participant level covariates, such as disease severity, also featured prominently in our literature review. If such covariates have a natural ordering, a deft meta-analysis of within‑trial interactions can be carried out assuming a linear trend across categories.^2^ Continuous variables such as age should not be categorised for statistical testing, because this loses power; worse, the choice of categorisation might affect the magnitude and statistical significance of the results.^12^ In the case of categorical covariates where a linear trend is not an appropriate assumption, it is possible to do a global test of interaction. This simply tests for some variation in treatment effect across participant subgroups without specifying its nature; but the risk of ecological bias in this context is unclear.

## Conclusions

Investigating associations between the effects of treatments or other interventions and participant level covariates can help identify who is most likely to benefit, and allow treatments to be targeted appropriately. In an era when such investigations are increasingly common, deft analysis and presentation should be the recommended approach for reliably informing clinical practice.

## Web Extra

Extra material supplied by the author

## Notes

Contributors: JFT and DJF conceived the initial idea and drafted the manuscript. DJF carried out the systematic literature review, performed the reanalysis of published data, and created the figures. He is the guarantor. JRC provided additional statistical expertise, and JRC and JFT helped revise the manuscript structure. All authors contributed to critical discussions of the ideas, commented on versions of the manuscript, and approved the final submission.
