# Supplementary material for: Meta-analytical methods to identify who benefits most from treatments: daft, deluded, or deft approach?
Source: BMJ. 2017 Mar 3;356:j573. doi: 10.1136/bmj.j573 (PMC5421441; doi:10.1136/bmj.j573)
Supplement: Supplementary file 1 — Appendix: Supplementary material [file fisd034884.ww.pdf]

WEB APPENDIX: SUPPLEMENTARY MATERIAL

WEB APPENDIX A: SYSTEMATIC LITERATURE SEARCH

A.1 SEARCH STRATEGY

Using MEDLINE (OVID version)

1	review.ab,ti.
2	review.pt.
3	meta analys?s.ab,ti.
4	meta analysis.pt.
5	meta analysis.kf.
6	overview.ab,ti.
7	1 or 2 or 3 or 4 or 5 or 6
8	letter.pt.
9	comment.pt.
10	editorial.pt.
11	protocol.ti.
12	analysis plan.ti
13	8 or 9 or 10 or 11 or 12
14	(participant or patient or person).ab,ti.
15	(individual adj 14).ab,ti.
16	(data$ or analys$ or records or pooled).ab,ti.
17	15 adj2 16
18	7 not 13
19	17 and 18
20	interaction$
21	trend$
22	subgroup$
23	covariate$
24	effect adj2 modifi$
25	20 or 21 or 22 or 23 or 24
26	19 and 25
27	limit 26 to yr="2011-2014"

N.B. The character $ represents a sequence of one or more letters or none; the character ? represents exactly one letter. “ab” = abstracts were searched; “ti” = titles were searched; “pt” = “publication type”; “kf” = “Keyword Heading Word”; “adj2” = “within two words of”; “yr” = year

A.2 ELIGIBLE RESULTS OF LITERATURE SEARCH

Notes:

- Reviews of diagnostic tests, prognostic modelling and gene–environment interaction studies were excluded
- If the same meta-analysis was published more than once, it was only counted once, but all relevant analyses and plots were considered.

w1. Alfredsson J et al. Impact of an invasive strategy on 5 years outcome in men and women with non-ST-segment elevation acute coronary syndromes. *Am Heart J* 2014; **168**(4): 522-9

w2. Ali S, Mealing S, Hawkins N et al. The use of individual patient-level data (IPD) to quantify the impact of pretreatment predictors of response to treatment in chronic hepatitis B patients. *BMJ Open* 2013; **3**: e001309.

w3. Arentz M et al. Use of anti-retroviral therapy in tuberculosis patients on second-line anti-TB regimens: a systematic review. *PLoS One* 2012; **7**(11): e47370

w4. Armaganijan LV et al. Are elderly patients at increased risk of complications following pacemaker implantation? A meta-analysis of randomized trials. *Pacing Clin Electrophysiol* 2012; **35**: 131-4

w5. Armstrong MJ et al. Safety and efficacy of liraglutide in patients with type 2 diabetes and elevated liver enzymes: individual patient data meta-analysis of the LEAD program. *Aliment Pharmacol Ther* 2013; **37**(2): 234-42

w6. Arrich J et al. Hypothermia for neuroprotection in adults after cardiopulmonary resuscitation. *Cochrane Database Syst Rev* 2012; **9**: CD004128

w7. Askie LM et al. Inhaled nitric oxide in preterm infants: an individual-patient data meta-analysis of randomized trials. *Pediatrics* 2011; **128**(4): 729-39

w8. Barta SK et al. Treatment factors affecting outcomes in HIV-associated non-Hodgkin lymphomas: a pooled analysis of 1546 patients. *Blood* 2013; **122**(19): 3251-62

w9. Bejon P et al. Efficacy of RTS,S malaria vaccines: individual-participant pooled analysis of phase 2 data. *Lancet Infect Dis* 2013; **13**(4): 319-27

w10. Ben-Shlomo Y et al. Aortic pulse wave velocity improves cardiovascular event prediction: an individual participant meta-analysis of prospective observational data from 17,635 subjects. *J Am Coll Cardiol* 2014; **63**(7): 636-46

w11. Bennett K et al. Cognitive behavioral therapy age effects in child and adolescent anxiety: an individual patient data metaanalysis. *Depress Anxiety* 2013; **30**(9): 829-41

w12. Berry DA et al. High-dose chemotherapy with autologous hematopoietic stem-cell transplantation in metastatic breast cancer: overview of six randomized trials. *J Clin Oncol* 2011; **29**(24): 3224-31

w13. Berry DA et al. High-dose chemotherapy with autologous stem-cell support as adjuvant therapy in breast cancer: overview of 15 randomized trials. *J Clin Oncol* 2011; **29**(24): 3214-23

w14. Blanchard P et al. Meta-analysis of chemotherapy in head and neck cancer (MACH-NC): a comprehensive analysis by tumour site. *Radiother Oncol* 2011; **100**(1): 33-40

w15. Blanchard P et al. Taxane-cisplatin-fluorouracil as induction chemotherapy in locally advanced head and neck cancers: an individual patient data meta-analysis of the meta-analysis of chemotherapy in head and neck cancer group. *J Clin Oncol* 2013; **31**(23): 2854-60

w16. Blood Pressure Lowering Treatment Trialists' Collaboration. Blood pressure-lowering treatment based on cardiovascular risk: a meta-analysis of individual patient data. *Lancet* 2014; **384**(9943): 591-8

w17. Bonati LH et al. Age modifies the relative risk of stenting versus endarterectomy for symptomatic carotid stenosis--a pooled analysis of EVA-3S, SPACE and ICSS. *Eur J Vasc Endovasc Surg* 2011; **41**(2): 153-8
w18. Boonacker CW et al. Adenoidectomy with or without grommets for children with otitis media: an individual patient data meta-analysis. *Health Technol Assess* 2014; **18**(5): 1-118
w19. Bower P et al. Influence of initial severity of depression on effectiveness of low intensity interventions: meta-analysis of individual patient data. *BMJ* 2013; **346**: f540
w20. Brand JS et al. Testosterone, sex hormone-binding globulin and the metabolic syndrome in men: an individual participant data meta-analysis of observational studies. *PLoS One* 2014; **9**(7): e100409
w21. Buyse M et al. Individual patient data meta-analysis of randomized trials evaluating IL-2 monotherapy as remission maintenance therapy in acute myeloid leukemia. *Blood* 2011; **117**(26): 7007-13
w22. Cardwell CR et al. Birth order and childhood type 1 diabetes risk: a pooled analysis of 31 observational studies. *Int J Epidemiol* 2011; **40**(2): 363-74
w23. Chiappini E et al. Use of combination neonatal prophylaxis for the prevention of mother-to-child transmission of HIV infection in European high-risk infants. *AIDS* 2013; **27**(6): 991-1000
w24. Cholesterol Treatment Trialists' (CTT) Collaboration. Lack of effect of lowering LDL cholesterol on cancer: meta-analysis of individual data from 175,000 people in 27 randomised trials of statin therapy. *PLoS One* 2012; **7**(1): e29849
w25. Cholesterol Treatment Trialists' (CTT) Collaborators. The effects of lowering LDL cholesterol with statin therapy in people at low risk of vascular disease: meta-analysis of individual data from 27 randomised trials. *Lancet* 2012; **380**(9841): 581-90
w26. Collaborative Group on Hormonal Factors in Breast Cancer. Menarche, menopause, and breast cancer risk: individual participant meta-analysis, including 118 964 women with breast cancer from 117 epidemiological studies. *Lancet Oncol* 2012; **13**(11): 1141-51
w27. Dangas GD et al. Meta-analysis of everolimus-eluting versus paclitaxel-eluting stents in coronary artery disease: final 3-year results of the SPIRIT clinical trials program (Clinical Evaluation of the Xience V Everolimus Eluting Coronary Stent System in the Treatment of Patients With De Novo Native Coronary Artery Lesions). *JACC Cardiovasc Interv* 2013; **6**(9): 914-22
w28. Di Leo A et al. HER2 and TOP2A as predictive markers for anthracycline-containing chemotherapy regimens as adjuvant treatment of breast cancer: a meta-analysis of individual patient data. *Lancet Oncol* 2011; **12**(12): 1134-42
w29. Donadini MP et al. Prognostic significance of residual venous obstruction in patients with treated unprovoked deep vein thrombosis: a patient-level meta-analysis. *Thromb Haemost* 2014; **111**(1): 172-9
w30. Early Breast Cancer Trialists' Collaborative Group (EBCTCG). Relevance of breast cancer hormone receptors and other factors to the effi cacy of adjuvant tamoxifen: patient-level meta-analysis of randomised trials. *Lancet* 2011; **378**: 771-84
w31. Early Breast Cancer Trialists' Collaborative Group (EBCTCG). Effect of radiotherapy after breast-conserving surgery on 10-year recurrence and 15-year breast cancer death: meta-analysis of individual patient data for 10,801 women in 17 randomised trials. *Lancet* 2011; **378**: 1707-16
w32. Farmer AJ et al. Meta-analysis of individual patient data in randomised trials of self monitoring of blood glucose in people with non-insulin treated type 2 diabetes. *BMJ* 2012; **344**: e486
w33. Fearon P et al. Services for reducing duration of hospital care for acute stroke patients. *Cochrane Database Syst Rev* 2012; **9**: CD000443
w34. Geersing GJ et al. Exclusion of deep vein thrombosis using the Wells rule in clinically important subgroups: individual patient data meta-analysis. *BMJ* 2014; **348**: g1340

w35. Gevers TJ et al. Young women with polycystic liver disease respond best to somatostatin analogues: a pooled analysis of individual patient data. *Gastroenterology* 2013; **145**(2): 357-65

w36. Ghio S et al. Prognostic implications of left ventricular dilation in patients with nonischemic heart failure: interactions with restrictive filling pattern and mitral regurgitation. *Congest Heart Fail* 2012; **18**(4): 198-204

w37. Gluud LL et al. Oral branched-chain amino acids have a beneficial effect on manifestations of hepatic encephalopathy in a systematic review with meta-analyses of randomized controlled trials. *J Nutr* 2013; **143**(8): 1263-8

w38. Gregson BA et al. Individual patient data subgroup meta-analysis of surgery for spontaneous supratentorial intracerebral hemorrhage. *Stroke* 2012; **43**(6): 1496-504

w39. Gupta V et al. Allogeneic, but not autologous, hematopoietic cell transplantation improves survival only among younger adults with acute lymphoblastic leukemia in first remission: an individual patient data meta-analysis. *Blood* 2013; **121**(2): 339-50

w40. Haasova M et al. The acute effects of physical activity on cigarette cravings: exploration of potential moderators, mediators and physical activity attributes using individual participant data (IPD) meta-analyses. *Psychopharmacology (Berl)* 2014; **231**(7): 1267-75

w41. Heneghan C et al. Self-monitoring of oral anticoagulation: systematic review and meta-analysis of individual patient data. *Lancet* 2012; **379**(9813): 322-34

w42. Houssami N et al. An individual person data meta-analysis of preoperative magnetic resonance imaging and breast cancer recurrence. *J Clin Oncol* 2014; **32**(5): 392-401

w43. Hoyo C et al. Body mass index in relation to oesophageal and oesophagogastric junction adenocarcinomas: a pooled analysis from the International BEACON Consortium. *Int J Epidemiol* 2012; **41**(6): 1706-18

w44. Hurwitz HI et al. Venous thromboembolic events with chemotherapy plus bevacizumab: a pooled analysis of patients in randomized phase II and III studies. *J Clin Oncol* 2011; **29**(13): 1757-64

w45. Huxley RR et al. A comparison of risk factors for mortality from heart failure in Asian and non-Asian populations: an overview of individual participant data from 32 prospective cohorts from the Asia-Pacific Region. *BMC Cardiovasc Disord* 2014; **14**: 61

w46. Jefferis J et al. Acute infective conjunctivitis in primary care: who needs antibiotics? An individual patient data meta-analysis. *Br J Gen Pract* 2011; **61**(590): e542-8

w47. Jeger RV et al. Early revascularization is beneficial across all ages and a wide spectrum of cardiogenic shock severity: A pooled analysis of trials. *Acute Card Care* 2011; **13**(1): 14-20

w48. Kengne AP et al. Fibrinogen and future cardiovascular disease in people with diabetes: aetiological associations and risk prediction using individual participant data from nine community-based prospective cohort studies. *Diab Vasc Dis Res* 2013; **10**(2): 143-51

w49. Laporte S et al. Individual patient data meta-analysis of enoxaparin vs. unfractionated heparin for venous thromboembolism prevention in medical patients. *J Thromb Haemost* 2011; **9**(3): 464-72

w50. MacPherson H et al. Characteristics of acupuncture treatment associated with outcome: an individual patient meta-analysis of 17,922 patients with chronic pain in randomised controlled trials. *PLoS One* 2013; **8**(10): e77438

w51. Mahmoodi BK et al. Association of mild to moderate chronic kidney disease with venous thromboembolism: pooled analysis of five prospective general population cohorts. *Circulation* 2012; **126**(16): 1964-71

w52. Martínez-Sellés M et al. Gender and survival in patients with heart failure: interactions with diabetes and aetiology. Results from the MAGGIC individual patient meta-analysis. *Eur J Heart Fail* 2012; **14**(5): 473-9

w53. Mathurin P et al. Corticosteroids improve short-term survival in patients with severe alcoholic hepatitis: meta-analysis of individual patient data. *Gut* 2011; **60**(2): 255-60

w54. Mauguen A et al. Hyperfractionated or accelerated radiotherapy in lung cancer: an individual patient data meta-analysis. *J Clin Oncol* 2012; **30**(22): 2788-97

w55. Mbuagbaw L et al. Mobile phone text messages for improving adherence to antiretroviral therapy (ART): an individual patient data meta-analysis of randomised trials. *BMJ Open* 2013; **3**(12): e003950

w56. Melsen WG et al. Attributable mortality of ventilator-associated pneumonia: a meta-analysis of individual patient data from randomised prevention studies. *Lancet Infect Dis* 2013; **13**(8): 665-71

w57. Meyer-Baron M et al. The neurobehavioral impact of manganese: results and challenges obtained by a meta-analysis of individual participant data. *Neurotoxicology* 2013; **36**: 1-9

w58. Miles DW et al. First-line bevacizumab in combination with chemotherapy for HER2-negative metastatic breast cancer: pooled and subgroup analyses of data from 2447 patients. *Ann Oncol* 2013; **24**(11): 2773-80

w59. Nelson JC et al. Moderators of outcome in late-life depression: a patient-level meta-analysis. *Am J Psychiatry* 2013; **170**(6): 651-9

w60. Nitsch D et al. Associations of estimated glomerular filtration rate and albuminuria with mortality and renal failure by sex: a meta-analysis. *BMJ* 2013; **346**: f324

w61. Nolan SJ et al. Phenytoin versus valproate monotherapy for partial onset seizures and generalised onset tonic-clonic seizures. *Cochrane Database Syst Rev* 2013; **8**: CD001769

w62. Ohashi Y et al. Meta-analysis of epoetin beta and darbepoetin alfa treatment for chemotherapy-induced anemia and mortality: Individual patient data from Japanese randomized, placebo-controlled trials. *Cancer Sci* 2013; **104**(4): 481-5

w63. Park DW et al. Frequency, causes, predictors, and clinical significance of peri-procedural myocardial infarction following percutaneous coronary intervention. *Eur Heart J* 2013; **34**(22): 1662-9

w64. Park DW et al. Long-term outcome of stents versus bypass surgery in diabetic and nondiabetic patients with multivessel or left main coronary artery disease: a pooled analysis of 5775 individual patient data. *Circ Cardiovasc Interv* 2012; **5**(4): 467-75

w65. Patti G et al. Clinical benefit of statin pretreatment in patients undergoing percutaneous coronary intervention: a collaborative patient-level meta-analysis of 13 randomized studies. *Circulation* 2011; **123**(15): 1622-32

w66. Pujol JL et al. Meta-analysis of individual patient data from randomized trials of chemotherapy plus cetuximab as first-line treatment for advanced non-small cell lung cancer. *Lung Cancer* 2014; **83**(2): 211-8

w67. Raja FA et al. Platinum versus platinum-combination chemotherapy in platinum-sensitive recurrent ovarian cancer: a meta-analysis using individual patient data. *Ann Oncol* 2013; **24**(12): 3028-34

w68. Ronellenfitsch U et al. Preoperative chemo(radio)therapy versus primary surgery for gastroesophageal adenocarcinoma: systematic review with meta-analysis combining individual patient and aggregate data. *Eur J Cancer* 2013; **49**(15): 3149-58

w69. Rossi A et al. Six versus fewer planned cycles of first-line platinum-based chemotherapy for non-small-cell lung cancer: a systematic review and meta-analysis of individual patient data. *Lancet Oncol* 2014; **15**(11): 1254-62

w70. Rothwell PM et al. Effect of daily aspirin on long-term risk of death due to cancer: analysis of individual patient data from randomised trials. *Lancet* 2011; **377**(9759): 31-41

w71. Rusinaru D et al. Relationship of serum sodium concentration to mortality in a wide spectrum of heart failure patients with preserved and with reduced ejection fraction: an individual patient data meta-analysis: Meta-Analysis Global Group in Chronic heart failure (MAGGIC). *Eur J Heart Fail* 2012; **14**(10): 1139-46

w72. Schuit E et al. Effectiveness of electronic fetal monitoring with additional ST analysis in vertex singleton pregnancies at >36 weeks of gestation: an individual participant data metaanalysis. *Am J Obstet Gynecol* 2013; **208**(3): 187

w73. Simes J et al. Aspirin for the prevention of recurrent venous thromboembolism: the INSPIRE collaboration. *Circulation* 2014; **130**(13): 1062-71

w74. Staples MP et al. Effectiveness of vertebroplasty using individual patient data from two randomised placebo controlled trials: meta-analysis. *BMJ* 2011; **343**: d3952

w75. Troughton RW et al. Effect of B-type natriuretic peptide-guided treatment of chronic heart failure on total mortality and hospitalization: an individual patient meta-analysis. *Eur Heart J* 2014; **35**(23): 1559-67

w76. Valle JW et al. Cisplatin and gemcitabine for advanced biliary tract cancer: a meta-analysis of two randomised trials. *Ann Oncol* 2014; **25**(2): 391-8

w77. Vanderstichel R et al. Predicting the effect of anthelmintic treatment on milk production of dairy cattle in Canada using an Ostertagia ostertagi ELISA from individual milk samples. *Prev Vet Med* 2013; **111**(1-2): 63-75

w78. Vollset SE et al. Effects of folic acid supplementation on overall and site-specific cancer incidence during the randomised trials: meta-analyses of data on 50,000 individuals. *Lancet* 2013; **381**(9871): 1029-36

w79. Wali RK et al. Efficacy and safety of carvedilol in treatment of heart failure with chronic kidney disease: a meta-analysis of randomized trials. *Circ Heart Fail* 2011; **4**(1): 18-26

w80. Yothers G et al. Outcomes among black patients with stage II and III colon cancer receiving chemotherapy: an analysis of ACCENT adjuvant trials. *J Natl Cancer Inst* 2011; **103**(20): 1498-506

w81. van den Boogaard NM et al. Prognostic profiles and the effectiveness of assisted conception: secondary analyses of individual patient data. *Hum Reprod Update* 2014; **20**(1): 141-51

w82. von Minckwitz G et al. Impact of treatment characteristics on response of different breast cancer phenotypes: pooled analysis of the German neo-adjuvant chemotherapy trials. *Breast Cancer Res Treat* 2011; **125**(1): 145-56

A.3 TRIALS INCLUDED IN REVIEW BY FEARON ET AL

w83. Anderson C et al. Home or hospital for stroke rehabilitation? Results of a randomized controlled trial. I: Health outcomes at 6 months. *Stroke* 2000;**31**:1024–31

w84. Donnelly M et al. Randomized controlled trial of an early discharge rehabilitation service: the Belfast community stroke trial. *Stroke* 2004;**35**(1):127–33

w85. Rudd AG et al. Randomised controlled trial to evaluate early discharge scheme for patients with stroke. *BMJ* 1997;**315**:1039–44

w86. Dey P et al. Home team trial (North Manchester General and Stepping Hill Hospitals). Unpublished data

w87. Mayo N et al. There's no place like home. An evaluation of early supported discharge for stroke. *Stroke* 2000;**31**:1016–23

w88. Rodgers H et al. Early supported hospital discharge following acute stroke: pilot study results. *Clinical Rehabilitation* 1997;**11**:280–7.

w89. Bautz-Holter E et al. Early supported discharge of patients with acute stroke: a randomized controlled trial. *Disability and Rehabilitation* 2002;**24**(7):348–55

w90. Widen Holmqvist L et al. A randomised controlled trial of rehabilitation at home after stroke in southwest Stockholm. *Stroke* 1998;**29**:591–7

w91. Askim T et al. Evaluation of an extended stroke unit service with early supported discharge for patients living in a rural community. A randomized controlled trial. *Clinical Rehabilitation* 2004;**18**:238–48.

WEB APPENDIX B: RE-ANALYSIS OF PUBLISHED DATA

B.1 EXTRACTION OF DATA FOR RE-ANALYSIS

As stated in the text, six studies from our literature review presented sufficient data to allow re-analysis, where “sufficient” implies that both an across-trial (*daft*) and a within-trial (*deft*) interaction were able to be estimated. In practice, all such examples that we identified presented covariates as binary or ordered categories, with treatment effect sizes and measures of variation or confidence limits presented by trial within each subgroup (see Figure 1a). This enabled re-analysis to be performed as described in Web Appendix B.2. One additional study[w11] appears to have suffered a misprint (the age category 8–11 years is missing from Figure 3) and hence was excluded.

From the six eligible studies, data were available to re-analyse 31 unique interactions between a binary treatment variable and a participant-level covariate, including multiple analyses using the same covariate but with different outcomes. Forest plots of the re-analyses are available from the authors on request.

B.2 STATISTICAL RE-ANALYSIS

Where the outcome was binary, such that raw count data was presented, the full IPD dataset could be reproduced and analyses carried out as described in Appendix B of Fisher et al[2]. Otherwise, the analyses were approximated as follows:

- **“Daft”:** Trial-level mean covariate values were approximated from the presented data. Overall treatment effect estimates were then regressed on these covariate values, but without the additive heterogeneity parameter usually included in meta-regression. This more closely approximates the across-trials component of most common “deluded” interaction methods.

- **“Deluded”:** A variance-weighted least-squares regression was performed of estimates of trial-within-subgroup treatment effects on subgroup identifier values. That is, for *i* trials and *k* subgroups, there would be *ik* observations. Under fixed-effects, this is equivalent to a variance-weighted least-squares regression of by-subgroup treatment effects on subgroup identifier values (that is, using *k* observations).

- **“Deft”:** Approximate trial-level estimates of covariate interaction were obtained from variance-weighted least-squares regressions of subgroup-specific treatment effects on subgroup identifier values for each trial separately. These estimates were then pooled across trials using standard fixed-effect inverse-variance meta-analysis.

WEB FIGURE:

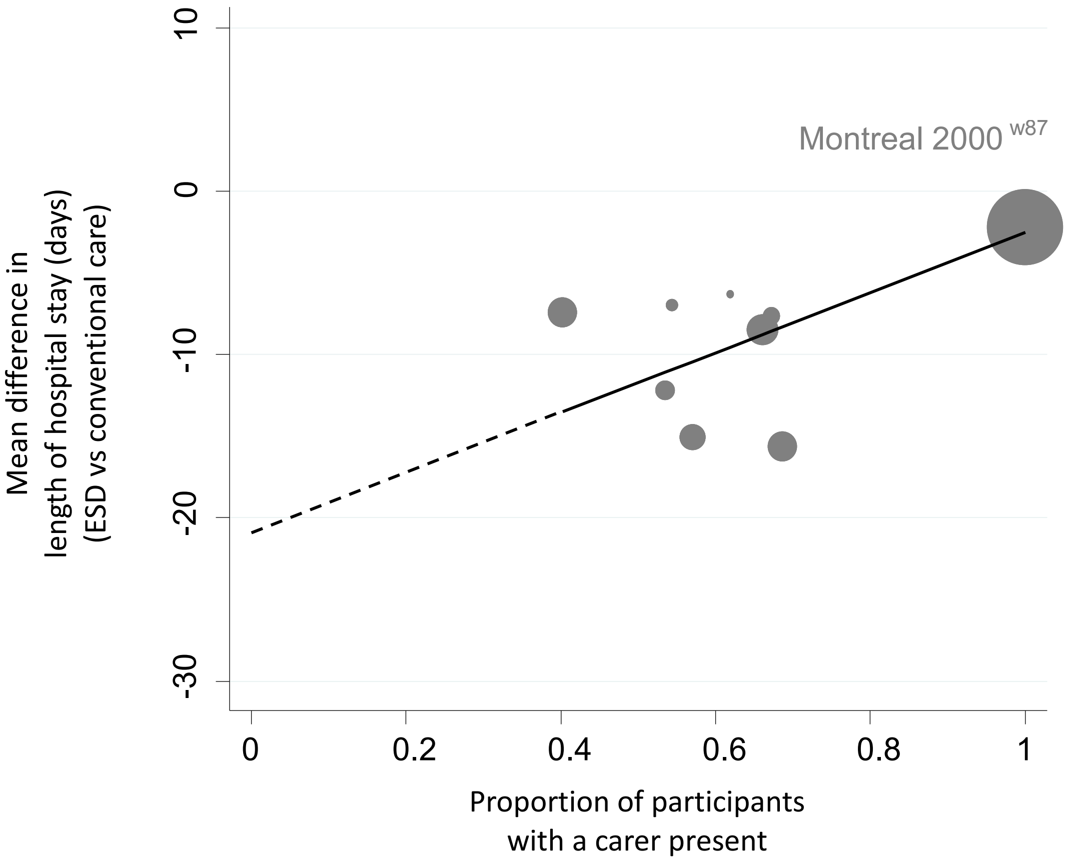

<u>Web Figure 1:</u> How the effect of an Early Supported hospital Discharge (ESD) strategy may vary by whether a carer is present[5] using a *daft* approach. Sizing of circles are in proportion to the inverse of the variance of the estimates. The line is the estimated slope from a meta-regression, where the solid part represents predictions within the range of observed data, and the dashed part is an extrapolation to a proportion of zero.
